# Prestimulation of CD2 confers resistance to HIV-1 latent infection in blood resting CD4 T cells

**DOI:** 10.1016/j.isci.2021.103305

**Published:** 2021-10-16

**Authors:** Sijia He, Jia Guo, Yajing Fu, Mark Spear, Chaolong Qin, Shuai Fu, Zongqiang Cui, Wenwen Jin, Xuehua Xu, Wanjun Chen, Hong Shang, Yuntao Wu

**Affiliations:** 1 NHC Key Laboratory of AIDS Immunology (China Medical University), National Clinical Research Center for Laboratory Medicine, The First Affiliated Hospital of China Medical University, Shenyang, Liaoning 110001, China; 2 Center for Infectious Disease Research, School of Systems Biology, George Mason University, Manassas, VA 20110, USA; 3 Mucosal Immunology Section, NIDCR, NIH, Bethesda, MD 20892, USA; 4 Chemotaxis Signal Section, Laboratory of Immunogenetics, National Institute of Allergy and Infectious Disease, NIH, Twinbrook Facility, Rockville, MD 20852, USA

**Keywords:** Immune response, Immunology, Virology

## Abstract

HIV-1 infects blood CD4 T cells through the use of CD4 and CXCR4 or CCR5 receptors, which can be targeted through blocking viral binding to CD4/CXCR4/CCR5 or virus-cell fusion. Here we describe a novel mechanism by which HIV-1 nuclear entry can also be blocked through targeting a non-entry receptor, CD2. Cluster of differentiation 2 (CD2) is an adhesion molecule highly expressed on human blood CD4, particularly, memory CD4 T cells. We found that CD2 ligation with its cell-free ligand LFA-3 or anti-CD2 antibodies rendered blood resting CD4 T cells highly resistant to HIV-1 infection. We further demonstrate that mechanistically, CD2 binding initiates competitive signaling leading to cofilin activation and localized actin polymerization around CD2, which spatially inhibits HIV-1-initiated local actin polymerization needed for viral nuclear migration. Our study identifies CD2 as a novel target to block HIV-1 infection of blood resting T cells.

## Highlights

• CD2 is highly expressed on human blood CD4 T cells, particularly memory T cells
• Prestimulation of CD2 rendered resting T cells highly resistant to HIV infection
• CD2 signaling activates cofilin and actin polymerization blocking HIV nuclear entry
• CD2 may serve as a novel target to inhibit HIV-1 infection of blood resting T cells

## Graphical abstract

**Figure:**
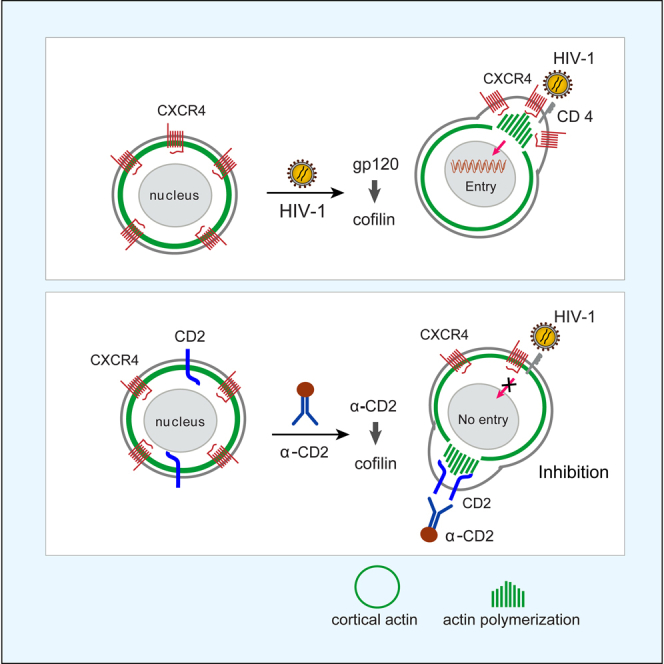

## Introduction

HIV infects and enters blood CD4 T cells through the use of CD4 (Dalgleish et al., 1984; Klatzmann et al., 1984) and the chemokine coreceptor, CXCR4 (Feng et al., 1996) or CCR5 (Alkhatib et al., 1996; Choe et al., 1996; Cocchi et al., 1995; Combadiere et al., 1996; Deng et al., 1996; Doranz et al., 1996; Dragic et al., 1996). Targeting HIV interaction with these receptors has been extensively explored for therapeutics (Qian et al., 2009). Mechanistically, HIV-1 entry can be blocked through the inhibition of viral attachment to cells, viral binding to CD4 or the chemokine coreceptors, and virus-cell fusion (Qian et al., 2009). Among many of the HIV entry inhibitors discovered, the fusion inhibitor, enfuvirtide (Wild et al., 1993), the CCR5 inhibitor, maraviroc (Dorr et al., 2005), and the anti-CD4 antibody, ibalizumab (Blair, 2020; Boon et al., 2002; Jacobson et al., 2009), have been successfully developed into clinical antiretroviral drugs.

HIV binding to the entry receptors not only mediates viral entry but also triggers signal transduction (Wu and Yoder, 2009). For example, HIV-1 binding to the chemokine coreceptor CXCR4 or CCR5 has been shown to trigger G-protein signaling critical for HIV latent infection of blood resting CD4 T cells (He et al., 2019; Yoder et al., 2008). Mechanistically, it has been suggested that the static cortical actin in blood resting T cells represents a barrier for viral entry and nuclear migration (Wang et al., 2012; Yoder et al., 2008). To overcome this restriction, HIV-1 relies on gp120 binding to CXCR4 or CCR5 to activate actin modulators such as cofilin to promote actin dynamics necessary for viral entry and nuclear migration (He et al., 2019; Spear et al., 2014; Vorster et al., 2011; Yoder et al., 2008). As such, chemotactic stimulation, such as stimulating resting CD4 T cells with the chemokines CCL19/CCL21, has been shown to activate the cofilin pathway and thereby promote HIV latent infection of resting T cells (Cameron et al., 2010). Conversely, blocking HIV-mediated G protein signaling and actin dynamics, using inhibitors such as pertussis toxin or the LIMK and Arp2/3 inhibitors, has also been shown to block HIV infection of blood resting T cells (Spear et al., 2014; Vorster et al., 2011; Yi et al., 2017; Yoder et al., 2008).

Cofilin is a member of the actin-depolymerizing factor/cofilin family of proteins that bind and depolymerize filamentous actin (F-actin) to regulate actin dynamics (Bernstein and Bamburg, 2010; Wu, 2013). In human T cells, cofilin regulates T cell motility for T cell migration and homing to lymphoid and non-lymphoid tissues. Cofilin is also an essential player in T cell activation, during which cofilin is activated by co-stimulatory signaling, including signaling from CD28 and integrins (e.g., LFA-1, α4β7), to promote actin polymerization for the stabilization of immunological synapse (Dustin and Cooper, 2000; Samstag et al., 2013).

Cluster of differentiation 2 (CD2) is also a co-stimulatory molecule abundantly expressed on the surface of blood CD4 T cells (Sanchez-Madrid et al., 1982). During T cell activation, CD2 interacts with adhesion molecules such as CD58/LFA-3 (lymphocyte function-associated antigen-3) to facilitate T cell activation (Bierer et al., 1989; Wang et al., 1999); ligation of CD2 activates cofilin to modulate actin cytoskeleton, enhancing T cell adhesion to antigen-presenting cells for T cell activation (Samstag et al., 1991, 1992, 1994). Given the demonstrated ability of CD2 to activate the cofilin pathway, we investigated potential impacts of CD2 signaling on HIV latent infection of resting CD4 T cells.

## Results

### Prestimulation of CD2 inhibits HIV-1 latent infection of resting CD4 T cells

**Figure 1:**
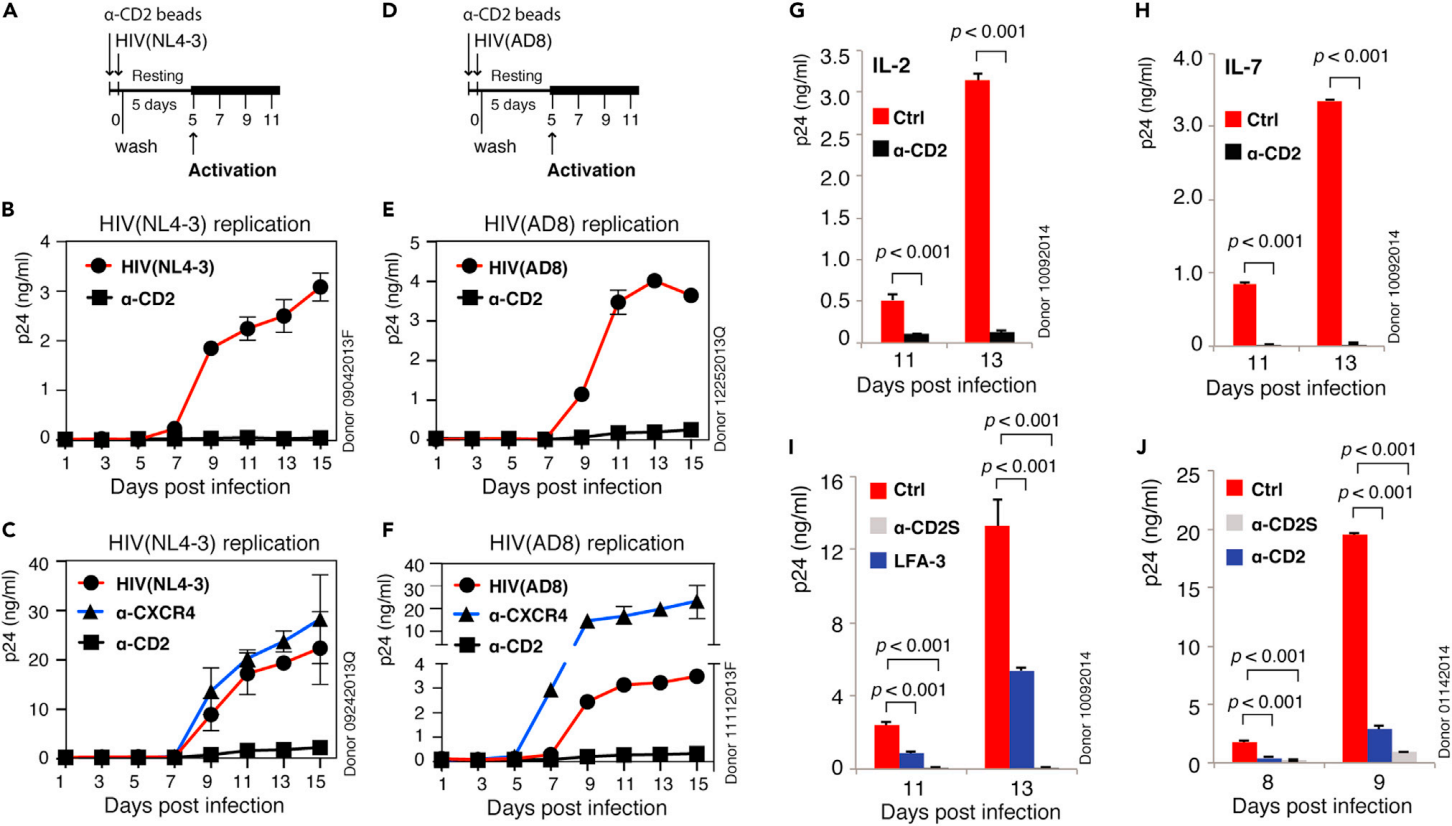
CD2 prestimulation inhibits HIV-1 latent infection of resting CD4 T cells. (A–C) Resting CD4 T cells were prestimulated with α-CD2 or α-CXCR4 beads and then infected with HIV-1(NL4-3) for 2 h. Cells were washed, cultured for 5 days, and then activated by α-CD3/CD28 beads to induce viral replication, which was measured by p24 release. The experiment was repeated in another two donors, and levels of p24 were quantified in triplicate in each donor. (D–F) Resting memory CD4 T cells were prestimulated with α-CD2 or α-CXCR4 beads and then infected with HIV-1(AD8) for 2 h. Cells were washed, cultured for 5 days, and then activated by α-CD3/CD28 beads to induce viral replication. Levels of p24 were quantified in triplicate. (G and H) Resting CD4 T cells were cultured in IL-2 or IL-7 for 3 days, prestimulated with α-CD2 beads, and then infected with HIV-1(NL4-3). Cells were washed, cultured for 5 days, and then activated by α-CD3/CD28 beads to induce viral replication. Levels of p24 were quantified in triplicate. (I) Resting CD4 T cells were prestimulated with soluble α-CD2 antibody (α-CD2s, 2 μg/mL) or recombinant human LFA-3 (2 μg/mL) for 1 h and then infected with HIV-1(NL4-3). Cells were similarly washed, cultured, and then activated at day 5. The experiment was repeated in two additional donors. Levels of p24 were quantified in triplicate. (J) Resting CD4 T cells were prestimulated with α-CD2 beads or soluble α-CD2 antibody (α-CD2s, 200 ng/mL) and then similarly infected and activated. The experiment was repeated in two additional donors. Levels of p24 were quantified in triplicate, and data are represented as mean ± SEM. Statistical significance was determined using two-tailed t test in Prism 7 (Graph Pad). Significance p values are indicated. See also Figures S1–S4.

To determine the effects of CD2 signaling on HIV infection of blood CD4 T cells, we first quantified CD2 expression on human blood resting CD4 T cells and found that both memory (CD45RO^+^) and naïve (CD45RO^−^) T cells expressed CD2, with memory T cells naturally expressing higher levels of surface CD2 (Iglesias-Ussel et al., 2013) (Figure S1). We further tested the effects of prestimulating resting T cells with an anti-CD2 antibody on HIV-1 latent infection. Prestimulation of resting CD4 T cells with an anti-CXCR4 antibody has been shown to trigger cofilin activation that enhances HIV-1 latent infection of resting T cells (Yoder et al., 2008). Surprisingly, when resting T cells were similarly prestimulated with the anti-CD2 antibody, we observed 90%–99% inhibition of HIV-1 latent infection of resting CD4 T cells (Figures 1A–1F); this CD2-mediated inhibition has been tested in a total of eight donors, and we observed strong inhibition in all donors tested. In contrast, prestimulation of CXCR4 enhanced HIV-1 latent infection (Figures 1C and 1F), as previously reported (Yoder et al., 2008).

Memory CD4 T cells are major targets of HIV-1 infection, and early depletion of memory CD4 T cells in the gastrointestinal tract by the CCR5 (R5)-utilizing T-tropic virus is a major cause of chronic immune activation in patients (Brenchley et al., 2004, 2006; Veazey et al., 1998). Thus, we also tested whether CD2 prestimulation can block latent infection of memory CD4 T cells by R5 virus. As shown in Figure 1 (1E and 1F), we also observed strong inhibition of HIV-1 latent infection of memory CD4 T cells by the R5 virus, HIV-1(AD8). Therefore, this anti-CD2-antibody-mediated inhibition of HIV-1 latent infection was observed in both X4 (NL4-3) and R5 (AD8) latent infection of resting and resting memory T cells, respectively. In addition, similar to the enhancement of X4 virus infection by prestimulation of CXCR4 (Figure 1C) (Yoder et al., 2008), CXCR4 prestimulation also enhanced the latent infection of memory CD4 T cells by the R5 virus (Figure 1F), which is in great contrast to the potent inhibition of HIV latent infection of memory T cells by CD2 prestimulation.

It is possible that this CD2-mediated inhibition may result from the inhibition of T cell activation. However, when the T cell activation markers CD25 and CD69 were analyzed, we did not observe inhibition of T cell activation (Figure S2). In addition, when CD2 stimulation was performed immediately following HIV-1 infection (CD2 poststimulation), it failed to inhibit HIV latent infection (Figure S3), suggesting that CD2 prestimulation likely triggered a cellular response that selectively blocks viral early infection steps.

Resting CD4 T cells circulate between the peripheral blood and lymphoid tissues. To further determine whether this CD2-mediated inhibition of HIV-1 early infection steps can be alleviated by lymphatic cytokines, we cultured resting CD4 T cells in IL-2 or IL-7 for 3 days and then latently infected cells with HIV-1. Cells were also similarly prestimulated with the anti-CD2 antibody. Again, we observed similar strong inhibition of HIV-1 latent infection by CD2 prestimulation (Figures 1G and 1H), demonstrating that these cytokines were not capable of overcoming CD2-mediated inhibition; previous studies have suggested that these cytokines are sufficient to permit low levels of HIV replication in resting CD4 T cells (Trinite et al., 2013; Unutmaz et al., 1999), partially overcoming cellular barriers present in resting T cells. Thus, this CD2-mediated block to HIV latent infection is likely different from known viral barriers in resting T cells (Pan et al., 2013).

We further tested whether prestimulation of resting CD4 T cells with the soluble CD2 ligand CD58 (LFA-3) can also block HIV-1 latent infection of resting CD4 T cells. As a control, we prestimulated resting CD4 T cells with a soluble anti-CD2 antibody. Again, we observed that prestimulation of resting CD4 T cells with either soluble LFA-3 or the anti-CD2 antibody blocked HIV-1 latent infection (Figures 1I, 1J, and S4), demonstrating that the anti-HIV activity from CD2 prestimulation did not result from possible peculiarities of the anti-CD2 antibody, but likely from intracellular signaling events initiated from CD2 binding by LFA-3 or the anti-CD2 antibody.

**Figure 2:**
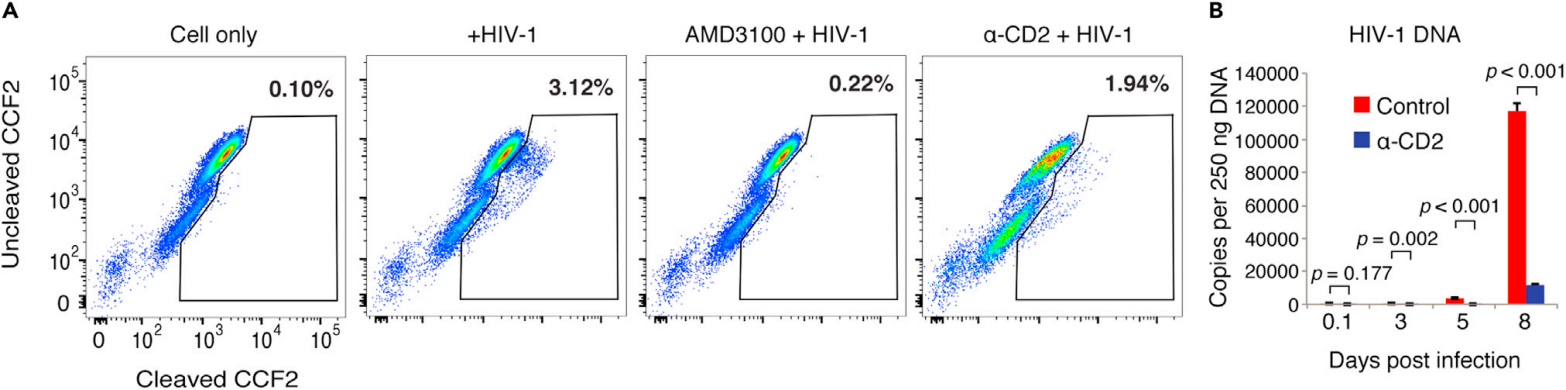
CD2 prestimulation inhibits HIV-1 entry and DNA accumulation in resting CD4 T cells (A) Resting CD4 T cells were prestimulated or not with α-CD2 beads and infected with a reporter virus, HIV-1(BlaM-Vpr). As a control, cells were also pretreated with AMD3100 and then infected. Viral entry was quantified by flow cytometry analysis of cleaved CCF2. The entry assay was repeated in two donors. (B) Resting CD4 T cells were prestimulated or not with α-CD2 beads, infected with an equal p24 level of HIV-1(NL4-3) for 2 h, washed, cultured for 5 days, and then activated with α-CD3/CD28 beads. Intracellular HIV late DNA was quantified by real-time PCR at indicated time points using an equal amount of total cellular DNA (250 ng). The experiment was repeated in two more donors, and samples were analyzed in triplicate and data are represented as mean ± SEM. Statistical significance was determined using two-tailed t test in Prism 7 (Graph Pad). Significance p values are indicated. See also Figure S5.

To further identify possible virological processes that are affected by CD2 prestimulation, we performed stepwise mapping of the viral early infection steps. An HIV-1 entry assay showed that CD2 prestimulation led to a partial inhibition of HIV-1 entry (from 3.12% to 1.94%) (Figure 2A). This partial inhibition is somewhat expected, given that CD2 is not a viral entry receptor and antibody-binding to CD2 may not completely block viral entry. The over 90% inhibition of HIV-1 latent infection of resting T cells by CD2 prestimulation, as we observed above (Figure 1), cannot be explained simply by CD2-mediated blockage of viral entry. Thus, we examined viral post-entry steps; when intracellular viral DNA was examined, we found that it was highly diminished (Figures 2B and S5). These results demonstrate that CD2 prestimulation inhibited viral early infection steps, including viral entry and post-entry accumulation of viral DNA in resting CD4 T cells.

### Prestimulation of CD2 activates the cofilin pathway

**Figure 3:**
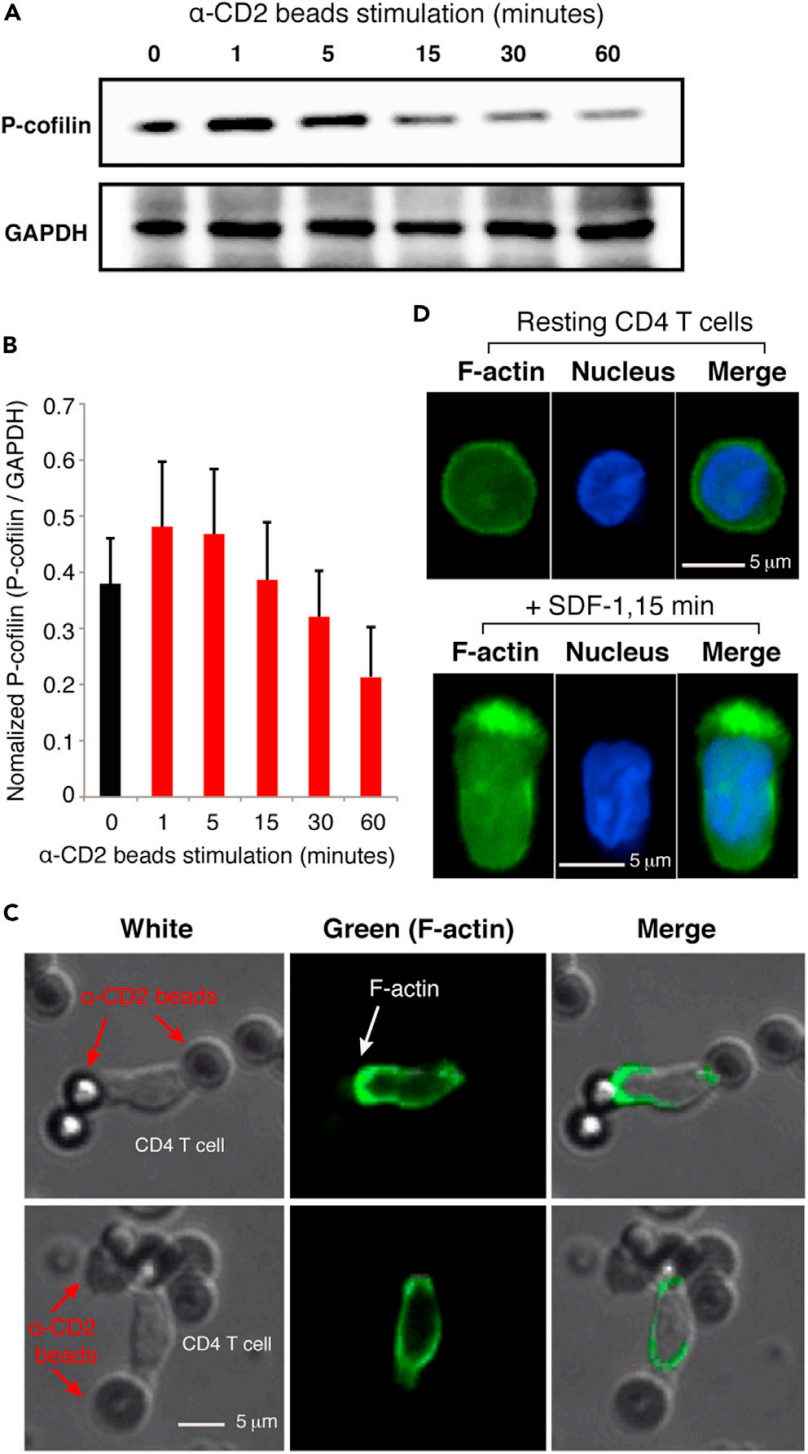
CD2 stimulation activates cofilin and actin dynamics in resting CD4 T cells. (A and B) Resting CD4 T cells were stimulated with α-CD2 antibody for a time course of 60 min and then lysed for western blot detection of cofilin phosphorylation. GAPDH was used as the loading control, and the relative ratio of cofilin phosphorylation is quantified in (B), which shows the normalized p-cofilin intensities on western blots, means ± SD from triplicate. (C) Resting CD4 T cells were first electroporated with pLifeAct-EGFP and then stimulated with α-CD2 beads. Actin polymerization was immediately monitored by live-cell fluorescence imaging microscopy. Shown are representative images from live cell imaging (supplemental information, Video S1). (D) Resting memory CD4 T cells were not stimulated or stimulated with SDF-1 (12.5 nM) for 5 min. Cells were fixed, stained with FITC-phalloidin for F-actin, and also stained with DAPI for nuclear DNA. Cells were then analyzed with confocal fluorescence microscopy. For unstimulated resting CD4 T cells, 1 of 24 cells showed spontaneous actin polymerization. Following stimulation, 25 of 29 cells showed polarized actin polymerization. See also Figure S6 and Video S1.

Prestimulation of resting CD4 T cells with anti-CXCR4 antibody enhances HIV-1 latent infection of resting CD4 T cells (Yoder et al., 2008); it has been shown that such stimulation leads to the activation of cofilin and localized actin activities, thereby facilitating viral entry and nuclear migration (Wang et al., 2012; Yoder et al., 2008). CD2 stimulation of T cells has also been shown to trigger cofilin activation (Samstag et al., 1991, 1992). However, given that CD2 prestimulation led to an opposite, strong inhibition of HIV-1 latent infection, we felt compelled to investigate the effects of CD2 stimulation on cofilin activation in resting CD4 T cells. As shown in Figure 3A, we observed that stimulation of CD2 with the anti-CD2 antibody triggered cofilin phosphorylation and dephosphorylation within minutes, as measured by immune staining and western blot of p-cofilin (Figure 3B). To confirm the result, we also developed an alternative approach to quantify p-cofilin by intracellular staining and flow cytometry (Figure S6). We observed a transient course of cofilin activation (dephosphorylation) and phosphorylation following CD2 stimulation, consistent with a previous report showing cofilin activation by CD2 stimulation (Samstag et al., 1994). We further examined actin dynamics following CD2 stimulation. For this purpose, resting CD4 T cells were transiently electroporated with a green fluorescent protein-tagged actin reporter construct (pLifeAct-EGFP). Cells were then stimulated with anti-CD2 antibody-coated magnetic beads and monitored with fluorescence microscopy live-cell imaging (Video S1). As summarized in the representative images (Figure 3C), anti-CD2 bead stimulation triggered intracellular actin polymerization around the bead-cell interface, demonstrating that actin polymerization occurred locally around the bound CD2 receptors. For comparison, stimulation of resting CD4 T cells with the CXCR4 ligand SDF-1 also triggered localized actin polymerization at the leading edge of migrating T cells enriched with CXCR4 (Figure 3D) (Guo et al., 2013; Yoder et al., 2008).

Video S1. Stimulation with anti-CD2-beads triggers actin polymerization around the bead-CD4 T cell junction, related to Figure 3 Blood resting CD4 T cells were purified, electroporated with pLifeAct-EGFP, and then stimulated with α-CD2 beads. Actin polymerization was monitored by live-cell fluorescence imaging microscopy, which uses the UltraView Vox confocal system (PerkinElmer, Co., contains cell culture chamber, Tokai Hit) with a Nikon Eclipse Ti-E microscope with a 60×, 1.4 NA oil-immersion objective lens. The images were captured with an EM-CCD (Hamamatsu C9100-14). Data were analyzed with Volocity 6.3.0. The green (F-actin) fluorescent field, the white field, and the merged field are shown (from left to right).

### CD2-initiated actin polymerization spatially competes with HIV-1 gp120-mediated actin polymerization

**Figure 4:**
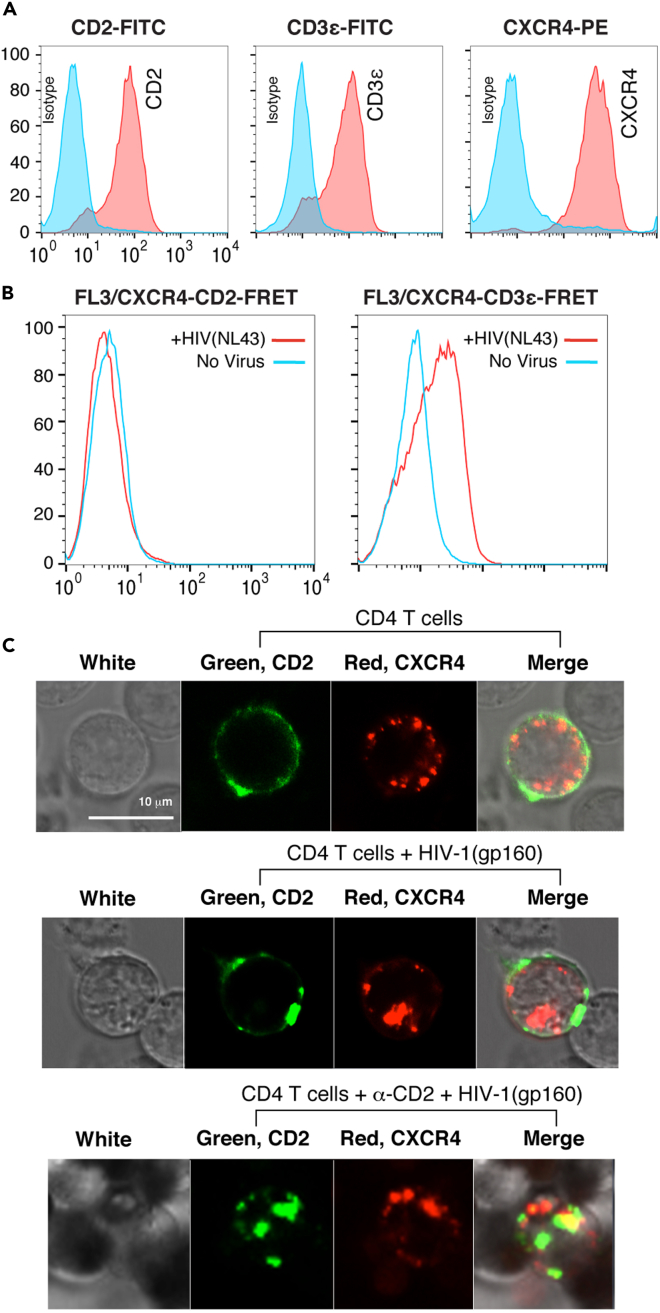
Lack of interaction and co-localization between CD2 and CXCR4 on CD4 T cells (A) Surface expression of CD2, CXCR4, and CD3ε. Jurkat CD4 T cells were stained by anti-CD2, -CXCR4, or CD3ε antibody and then analyzed by flow cytometry. (B) Cells were also stimulated with HIV-1(NL4-3). FRET signal between CD2 and CXCR4 was quantified by flow cytometry, using FRET between CXCR4 and CD3ε as a control. (C) Cells were also electroporated with a CD2-GFPSpark vector (green) and a CXCR4-OFPSpark vector (red) and then stimulated with HIV-1(gp160). CD2 and CXC4 colocalization before (top panel) and during HIV-1 infection (middle panel), and following α-CD2 bead prestimulation and HIV-1 infection (bottle panel), was analyzed by confocal microscopy. See also Figure S7.

Given the opposite effects of CXCR4 and CD2 prestimulation on HIV-1 latent infection as demonstrated above (Figure 1) (Wang et al., 2012; Yoder et al., 2008), we speculated that CD2-initiated cofilin and actin polymerization may spatially compete with the HIV-1/CXCR4-initiated cofilin and actin activity that is needed for viral entry and nuclear migration. To test this hypothesis, we first examined the possible effects of CD2 stimulation on the down-regulation of CD4 and CXCR4 but did not observe CD4 or CXCR4 downregulation in response to CD2 stimulation (Figure S7). We then examined possible CD2-CXCR4 interaction during viral entry. We performed a fluorescence resonance energy transfer (FRET) assay using a human CD4 T cell, Jurkat, which expresses both CXCR4 and CD2 (Figure 4A). FRET occurs when specific pairs of chromophores come within approximately 10 nm of each other (Stryer, 1978). As a control, we also examined a surface glycoprotein, the CD3ε chain, which is expressed on Jurkat T cells (Figure 4A); stimulation of CXCR4 with SDF-1 has been shown to trigger CXCR4-CD3ε interaction and their co-clustering on T cells (Kumar et al., 2006). For detecting possible interaction and FRET between CXCR4 and CD2 or between CXCR4 and CD3ε, we used a PE-labeled anti-human CXCR4 antibody (emission peak at 574 nm) and an APC-labeled anti-human CD2 antibody (emission peak at 660 nm) or an APC-labeled anti-human CD3ε antibody. Cells were first labeled at low temperature with the PE-anti-CXCR4 antibody and the APC-anti-CD2 antibody or with the PE-anti-CXCR4 antibody and the APC-anti- CD3ε antibody and then left unstimulated or stimulated with HIV particles. HIV binding to CXCR4 may induce the formation of the CXCR4-CD2 complexes or the CXCR4-CD3ε complexes (Kumar et al., 2006), which can be endocytosed into endosomes for detection. We also used a cold acid wash to remove cell-surface antibodies so that intracellular FRET signals from these complexes were selectively quantified. As shown in Figure 4B, stimulation of Jurkat T cells with HIV-1 particles triggered FRET between CXCR4 and CD3ε, suggesting that, as expected (Kumar et al., 2006), HIV gp120-CXCR4 interaction induced co-clustering of CXCR4 and CD3ε (Figure 4B). However, we did not observe HIV-mediated FRET between CXCR4 and CD2 (Figure 4B), suggesting that CD2 may not co-localize and interact with CXCR4 during HIV binding and entry. CXCR4 and CD2 are likely localized differently on the plasma membrane of CD4 T cells. To further confirm this speculation, we directly observed CD2 and CXCR4 localization during gp120 binding. For this purpose, we transiently transfected fluorescently tagged CD2 and CXCR4 into T cells to monitor receptor dynamics. We stimulated the cells with HIV-1(gp120) virion particles, and again, as shown in Figure 4C, we observed that HIV-1 gp120 binding induced CXCR4 clustering but CD2 was largely excluded from co-localization with CXCR4. Conversely, prestimulation of cells with anti-CD2 beads induced CD2 clustering, but CXCR4 were excluded from co-localization with CD2. Our results are consistent with previous findings that HIV-1 gp120 induces actin-dependent co-clustering of CXCR4 (Barrero-Villar et al., 2009) and CD2 signaling induces actin-dependent clustering of CD2 into distinct plasma membrane microdomains (Kaizuka et al., 2009; Li et al., 1996). However, our data clearly demonstrate that the CXCR4 and CD2 microdomains distributed differently and did not co-localize on the plasma membrane during HIV entry (Figure 4C). Thus, CD2-initiated actin polymerization around CD2 can spatially compete and interfere with HIV-1 gp120-mediated actin polymerization around CXCR4 at the site of viral entry.

### Prestimulation of CD2 inhibits HIV nuclear migration

**Figure 5:**
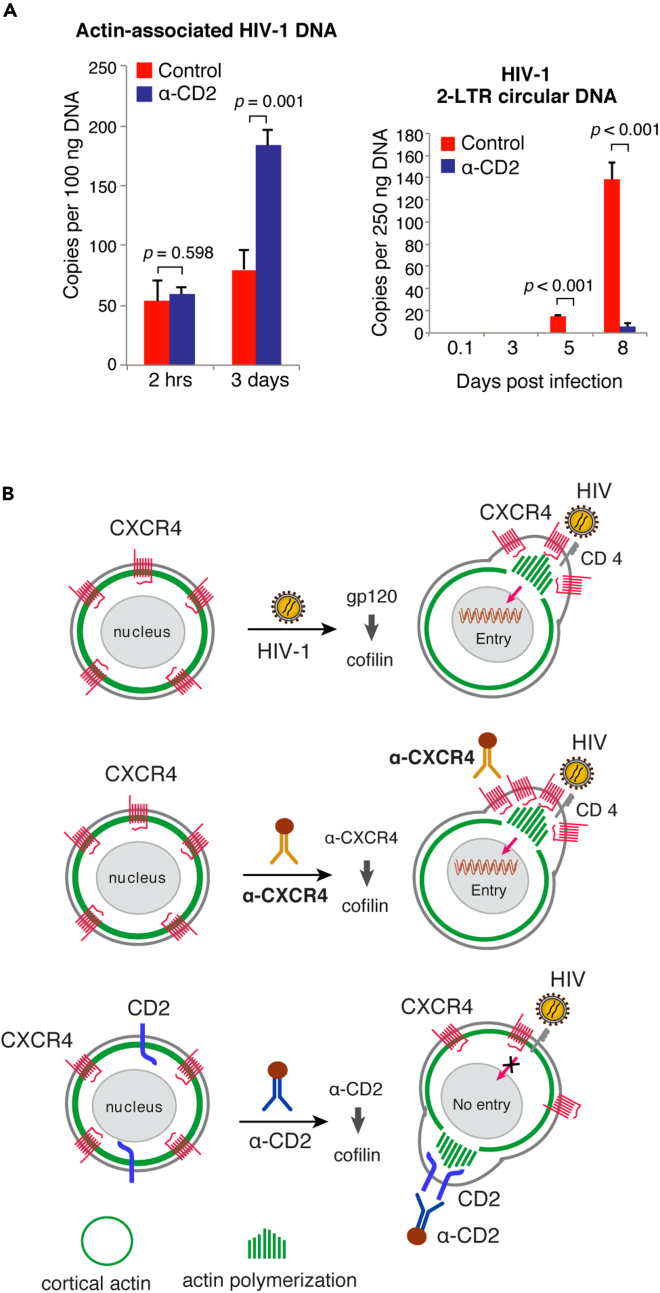
CD2 signaling inhibits HIV-1 nuclear entry (A) Resting CD4 T cells were prestimulated with α-CD2 beads and infected with HIV-1(gp160). Actin cytoskeleton-associated viral DNA was quantified (left), and viral nuclear 2-LTR circles were also quantified by real-time PCR. (B) Model of CD2 signaling in blocking HIV-1 latent infection of CD4 T cells. CD2 signaling spatially inhibits viral entry and nuclear migration through triggering cofilin activation and actin polymerization around CD2, which does not spatially co-localize with CXCR4. CD2 signaling may competitively inhibit HIV-1-mediated CXCR4 signaling needed for viral entry and nuclear migration. Samples were analyzed in triplicate, and data are represented as mean ± SEM. Statistical significance was determined using two-tailed t test in Prism 7 (Graph Pad). Significance p values are indicated. See also Figure S8.

Previous studies have demonstrated that HIV-1-mediated actin dynamics is required for promoting viral nuclear migration in resting CD4 T cells (Yoder et al., 2008). As shown above (Figure 2), CD2 prestimulation only partially inhibited HIV-1 entry into resting CD4 T cells. However, HIV latent infection of resting T cells were highly diminished by CD2 prestimulation (Figure 1), suggesting that the residual viral particles entering cells did not contribute to the productive viral infection process. We speculated that these residual particles are likely blocked from further nuclear entry as a result of CD2-induced actin dynamics, which may inhibit the gp120/CXCR4-initiated local actin dynamics needed for viral nuclear migration (Yoder et al., 2008). To test this hypothesis, we performed cellular fractionation and quantified actin-associated viral DNA in the cytoplasm and viral DNA in the nucleus. The actin cytoskeleton has been shown to be an active site for viral reverse transcription (Bukrinskaya et al., 1998). We quantified viral nuclear DNA using 2-LTR circles as a marker (Bukrinsky et al., 1992; Taddeo et al., 1994; Yoder et al., 2008). As shown in Figure 5A, we observed an increase in actin-associated viral DNA in the cytoplasm even with a lower viral entry in the CD2-prestimulated cells. However, contrary to the actin-associated viral DNA, the nuclear 2-LTR circles were highly diminished in the CD2-prestimulated cells (Figures 5A and S8), suggesting that CD2 prestimulation likely led to the retention of the viral pre-integration complex (PIC) in the cortical actin meshwork; the much higher amounts of viral nuclear DNA found in the CD2 un-stimulated control cells suggest successful viral PIC nuclear translocation for the latent infection of resting T cells.

## Discussion

In this article, we describe a novel mechanism by which HIV-1 nuclear migration can be blocked through targeting a non-entry receptor, CD2. We demonstrate that prestimulation of resting CD4 T cells with the anti-CD2-antibody triggers localized cofilin activation and actin polymerization around CD2, which clusters into distinctive microdomains on the plasma membrane (Kaizuka et al., 2009; Li et al., 1996); this anti-CD2 antibody-initiated actin polymerization and receptor clustering localize differently from the HIV-1 gp120/CXCR4-mediated receptor clustering at the site of viral entry. Therefore, CD2 signaling may competitively inhibit HIV-1 entry and nuclear migration required for the establishment of viral latency in resting CD4 T cells (Figure 5B). Indeed, our study demonstrated that CD2 prestimulation led to the diminishment of HIV latent infection of resting T cells (Figure 1). Mechanistic studies further demonstrated that there was an approximately 40% reduction in viral entry in CD2 prestimulated cells (Figure 2A). However, the reduction in viral entry cannot be used to simply explain the diminishment of viral latent infection. Thus, additional post-entry inhibition likely exists. Our conclusion of additional blockage at the step of viral post-entry nuclear migration came from multiple results: (1) The post-entry stimulation of resting CD4 T cells with CD2 did not block HIV latent infection (Figure S3), which is in great contrast to the strong inhibition of HIV by CD2 prestimulation (Figure 1), suggesting that CD2-mediated inhibition is likely limited to viral early infection steps such as viral entry and nuclear migration. (2) We directly observed a strong inhibition of the accumulation of 2-LTR circles in CD2 prestimulated cells (Figure 5A); 2-LTR circles are a common marker used to quantify HIV nuclear entry (Bukrinsky et al., 1992; Butler et al., 2001; Sloan and Wainberg, 2011). (3) Consistently, we also observed a transient increase in the actin-associated cytosolic viral DNA in CD2 prestimulated cells, which is in contrast to the decrease of the nuclear 2-LTR circles (Figure 5A). Together, these results suggest that, in CD2 prestimulated cells, HIV DNA is likely blocked from nuclear entry, and temporarily retained in the cortical actin layer, and subsequently degraded.

Our observation of the surprise increase of actin-associated viral DNA in the CD2-prestimulated cells, even with lower viral entry, suggests that the cortical actin barrier is also an active site for viral reverse transcription (Bukrinskaya et al., 1998; Yoder et al., 2008). The accumulation of actin-associated viral DNA, as seen in the CD2-prestimulated cells, resembles that observed in the cofilin knockdown CD4 T cells (Yoder et al., 2008); cofilin knockdown reduces actin depolymerization, leading to an increase in viral DNA synthesis but a decrease in viral nuclear migration (Yoder et al., 2008). These results suggest that viral particles retained in the cortical actin layer are transiently active for reverse transcription but blocked from further nuclear translocation by the absence of active actin treadmilling (Yin et al., 2020). The actin-associated viral DNA also appears to be labile (Zack et al., 1990) and eventually diminished with time in resting T cells (Figure 2). Thus, viral nuclear migration is likely a critical step for maintaining the viral pre-integration complex in resting T cells that is needed for the establishment of viral persistence (Wu and Marsh, 2001).

Our results are consistent with previous studies that underline the importance of HIV-1 gp120-mediated chemokine coreceptor signaling for viral latent infection of resting T cells (Cameron et al., 2010; Yoder et al., 2008). In contrast to CD2 signaling, prestimulation of resting CD4 T cells with anti-CXCR4 antibody, with the chemokines CCL19/CCL21, or with IP-10 (CXCL10) has been shown to promote HIV-1 latent infection of resting CD4 T cells (Cameron et al., 2010; Saleh et al., 2007; Wang et al., 2021). Interestingly, the CCL19/CCL21 receptor, CCR7, has been shown to form a heterodimer with CXCR4 (Hayasaka et al., 2015; Levoye et al., 2009), suggesting that stimulation of CCR7 likely initiates localized actin activity around CXCR4 at the site of viral entry, and thereby may synergize with gp120/CXCR4-initiated actin dynamics to promote viral entry and nuclear migration. In addition, the IP-10 receptor CXCR3 has also been shown to form functional heteromeric complexes with CXCR4 (Watts et al., 2013). IP-10 has been found to activate the cofilin pathway and promote HIV latent infection of resting memory CD4 T cells (Wang et al., 2021), likely by a similar mechanism through synergizing with HIV-mediated CXCR4 signaling (Yoder et al., 2008).

In addition to triggering cofilin activation and actin polymerization, CD2 stimulation may also induce other cellular factors restricting HIV-1 latent infection of resting T cells. However, CD2 stimulation-mediated inhibition of HIV-1 latent infection occurs only before, not after, viral entry (Figures 1 and S3). Given that CD2 post-entry stimulation did not inhibit cycles of viral replication, it is unlikely that CD2 stimulation induces unknown restriction factors blocking HIV latent infection.

Our discovery of the anti-HIV activity of CD2 signaling may offer new therapeutics to prevent HIV latent infection of blood CD4 T cells. Anti-CD4 antibody-mediated blockage of viral entry has been developed and currently approved for the clinical management of HIV infection (Blair, 2020; Boon et al., 2002; Jacobson et al., 2009). Our studies demonstrate that the anti-CD2 antibody can also potently block viral entry and nuclear entry by a different mechanism (Figure 5B). In a mouse model, the CD2 receptor was found to be functionally dispensable for the development and function of T cells; the homozygous CD2 mutant mice are healthy and have an apparently normal complement of lymphocytes (Killeen et al., 1992). These previous studies provide a rationale for targeting CD2 to block HIV-1 infection of blood CD4 T cells.

### Limitations of the study

There are several limitations to the present study. First, although prestimulation of CD2 on blood resting CD4 T cells blocks HIV entry and nuclear migration, such inhibition may only be observed in primary resting CD4 T cells but not in transformed T cell lines or activated T cells. It has been shown that the cortical actin in resting T cells represents a unique restriction to HIV latent infection (Yoder et al., 2008). HIV relies on Env-mediated chemokine coreceptor signaling to activate cofilin and actin dynamics facilitating viral nuclear entry (Yoder et al., 2008). This viral requirement for coreceptor signaling can only be observed in blood resting CD4 T cells but not in transformed cell lines (Cocchi et al., 1996; Wu and Yoder, 2009). Thus, it is possible that the competition between HIV-mediated coreceptor signaling and CD2 signaling may occur only in resting T cells but not in transformed cell lines or activated T cells. Second, the inhibitory phenotype of CD2 stimulation is observed *in vitro* in cell culture conditions and needs to be confirmed *in vivo* in animal models. Finally, novel small molecule inhibitors of CD2 may need to be developed and tested for inhibiting HIV latent infection of blood CD4 T cells.

## STAR★Methods

### Key resources table

**Table:** 

REAGENT or RESOURCE	SOURCE	IDENTIFIER
**Antibodies**		

Purified CD2 (clone RPA-2.10)	BD Biosciences	Cat#555323
Purified CXCR4 (clone 12G5)	BD Biosciences	Cat#555971
Purified CD3 (clone HIT3α)	BD Biosciences	Cat#555336
Purified CD28 (clone CD28.2)	BD Biosciences	Cat#555725
PE-CD25 (clone M-A251)	BD Biosciences	Cat#555432
PE-CD69 (clone FN50)	BD Biosciences	Cat#557050
FITC-CD2 (clone RPA-2.10)	Biolegend	Cat#300206
FITC-CD3ε (clone UCHT1)	BD Biosciences	Cat#555332
PE-CXCR4 (clone 12G5)	BD Biosciences	Cat#555974
APC-CD2 antibody (clone RPA-2.10)	BD Biosciences	Cat# 560642
PE-CXCR4 antibody (clone 12G5)	Biolegend	Cat# 306506
APC-CD3ε antibody (clone UCHT1)	BD Biosciences	Cat# 555335
P-cofilin antibody (Rabbit)	Cell signaling	Cat#3313
Anti-rabbit IgG, HRP-linked Antibody	Cell Signaling	Cat#7074
Alexa Fluor 488 chicken anti-Rabbit Molecular Probes	Invitrogen	Cat#A21441

**Bacterial and virus strains**		

HIV-1(NL4-3)	NIH HIV Reagent Program	ARP-114
HIV-1(AD8)	Englund et al., 1995	N/A

**Biological samples**		

Human blood	Healthy adult	N/A

**Chemicals, peptides, and recombinant proteins**		

Recombinant Human IL-2	PeproTeck	Cat#200-02
Recombinant Human IL-7	PeproTeck	Cat#200-07
Recombinant Human CD58/LFA-3	R&D Systems	Cat#1689-CD

**Critical commercial assays**		

LiveBLAzer™ FRET-B/G Loading Kit with CCF2-AM	Invitrogen	Cat#K1032
Lipofectamine 2000 Transfection Reagent	Invitrogen	Cat#11668-019
Dynabeads Pan Mouse IgG	Invitrogen	Cat#11042

**Deposited data**		

Raw and analyzed data	This paper	Lab notebook

**Experimental models: cell lines**		

Jurkat CD4 T cell	NIH HIV Reagent Program	ARP-177
HEK293T cell	ATCC	CRL-3216

**Oligonucleotides**		

HIV DNA forward primer 5’LTR-U5:5’- AGAT CCCTCAGACCCTTTTAGTCA-3’	This paper	N/A
HIV DNA reverse primer 3’ gag: 5’- TTCGCTTTCAAGTCCCTGTTC-3’	This paper	N/A
HIV DNA probe FAM-U5/gag:5'-(FAM)- TGT GGAAAATCTCTAGCAGTGGCGCC-(BHQ)-3’	This paper	N/A
HIV 2-LTR primer MH535: 5’-AACTAGGGAACCCACTGCTTAAG-3’	This paper	N/A
HIV 2-LTR primer MH536:5’-TCCACAGAT CAAGGATATCTTGTC-3’	This paper	N/A
HIV 2-LTR probe MH603:5'-(FAM)-ACACT ACTTGAAGCACTCAAGGCAAGC TTT-(BHQ)-3'	This paper	N/A

**Recombinant DNA**		

pNL4-3 proviral DNA	NIH HIV Reagent Program	ARP-114
pAD8 proviral DNA	Englund et al., 1995	N/A
pCMV4-BlaM-Vpr	Dr. Warner C. Greene	N/A
pAdVAntage	Promega	Cat# E1711
pLifeAct-EGFP	Addgene	58470
pCMV3-CD2-C-GFPSpark	Sino Biological	Cat#HG10982-ACG
pCMV3-CXCR4-C-OFPSpark	Sino Biological	Cat#HG11325-ACR

Software and algorithms		

GraphPad Prism	GraphPad Software Inc.	https://www.graphpad.com
FlowJo	Becton Dickinson	https://www.flowjo.com
Adobe Photoshop	Adobe	https://www.adobe.com/products/photoshop.html
Adobe Illustrator	Adobe	https://www.adobe.com/products/illustrator.html

### Resource availability

#### Lead contact

Further information and request for resources and reagents should be directed to and will be fulfilled by the Lead Contact, Yuntao Wu (ywu8@gmu.edu).

#### Materials availability

This study did not generate new unique reagents

### Experimental model and subject details

The study involved the use of human peripheral blood from twenty adult donors. Donor gender identity information was kept confidential per protocols and there is no scientific basis for gender preference in donor selection. Informed consent was obtained from all subjects. All protocols involving human subjects were reviewed and approved by the George Mason University institutional review board or by the Ethics Review Committee of China Medical University.

### Method details

#### Viruses and HIV-1 infection of blood resting CD4 T cells

Blood resting CD4 T cells were purified from peripheral blood of HIV-negative donors by two rounds of negative selection as previously described (Wu and Marsh, 2001; Yoder et al., 2008). Briefly, for the first-round depletion, monoclonal antibodies against human CD14, CD56, and HLA-DR, DP, and DQ (BD Biosciences) were used. For the second-round depletion, monoclonal antibodies against human CD8, CD11b, and CD19 (BD Biosciences) were used. Antibody-bound cells were depleted using Dynabeads Pan Mouse IgG (Thermo Fisher Scientific). For further negative selection of the memory CD4 T cell subsets, monoclonal antibody against CD45RA (0.02 μl per million cells) (BD Biosciences) was added during the second round of depletion. Purified cells were cultured in RPMI-1640 medium supplemented with 10% heat-inactivated fetal bovine serum (Invitrogen, Carlsbad, CA), penicillin (50 U/ml) (Thermo Fisher Scientific), and streptomycin (50 μg/ml) (Thermo Fisher Scientific). Virus stocks of HIV-1(NL4-3) and HIV-1(AD8) (Englund et al., 1995) were prepared by transfection of HEK293T cells with proviral DNA using Lipofectamine 2000 (Thermo Fisher Scientific), as described previously (He et al., 2019; Yoder et al., 2008). Briefly, 3 x 10^6^ HEK293T cells were cultured in 10 cm petri dish overnight, and then transfected with 20 μg of proviral DNA mixed with 60 μl of Lipofectamine 2000. Cells were transfected for 6 hours, washed, and cultured for 48 hours in DMEM medium supplemented with 10% heat-inactivated fetal bovine serum (Invitrogen, Carlsbad, CA), penicillin (50 U/ml) (Thermo Fisher Scientific), and streptomycin (50 μg/ml) (Thermo Fisher Scientific). Viruses in the supernatant were harvested and filtered through a 0.45-μm nitrocellulose membrane. Levels of p24 in the viral supernatant were measured by using an in-house p24 ELISA kit. Single cycle HIV-1(gp160) was prepared as described previously (Yu et al., 2009). Briefly, 3 x 10^6^ HEK293T cells were cultured in 10 cm petri dish overnight, and then transfected with 10 μg of pNL4-3(KFS) DNA and 10 μg of pNLΔψEnV mixed with 60 μl of Lipofectamine 2000. Cells were transfected for 6 hours, washed, and cultured for 48 hours in DMEM medium supplemented with 10% heat-inactivated fetal bovine serum (Invitrogen, Carlsbad, CA), penicillin (50 U/ml) (Thermo Fisher Scientific), and streptomycin (50 μg/ml) (Thermo Fisher Scientific). Viruses in the supernatant were harvested and filtered through a 0.45-μm nitrocellulose membrane. Levels of p24 in the viral supernatant were measured by using an in-house p24 ELISA kit. For infection, CD4 T cells were incubated with viruses for 2 hours, washed twice with medium to remove unbound virus, and then cultured in fresh medium (10^6^ cells per ml) for 5 days. Cells were activated at day 5 with anti-CD3/CD28 conjugated beads at 4 beads per cell as previously described (Wu and Marsh, 2001; Yoder et al., 2008). Culture supernatant was taken daily or every two days after stimulation and used for p24 ELISA.

#### Conjugation of antibodies to magnetic beads

Monoclonal antibodies against human CD2 (clone RPA-2.10), CXCR4 (clone 12G5), CD3 (clone HIT3α), or CD28 (clone CD28.2) were purchased commercially (BD Biosciences). For conjugation, 10 μg of antibodies was conjugated with 4 x 10^8^ Dynal beads (Thermo Fisher Scientific) for 30 minutes at room temperature. The magnetic beads were washed with PBS-0.5% BSA and resuspended in 1 ml of PBS-0.5%BSA.

#### Stimulation of resting CD4 T cells

Resting CD4 T cells were stimulated with antibodies or LFA-3 (CD58). For pre-stimulation with the anti-CD2 antibody-conjugated magnetic beads, resting CD4 T cells (10^6^ cells) were mixed with the beads (2 beads per cell), cultured at 37°C overnight, and then infected with HIV-1. For stimulation with soluble anti-CD2 antibody (clone RPA-2.10) (BD Biosciences) or recombinant human CD58 (R&D Systems), resting CD4 T cells (10^6^ cells) were treated at the indicated concentrations for 1 hour and then infected with HIV-1.

#### Surface staining of receptors on resting CD4 T cells and Jurkat T cells

For measuring surface expression of CD2 on naïve CD4 T cells and resting memory CD4 T cells, resting CD4 T cells (10^6^ cells) were stained with FITC-conjugated anti-human CD2 antibody (clone RPA-2.10) (Biolegend) and PE-Cy5-conjugated anti-Human CD45RO antibody (clone UCHL1) (BD Biosciences). For quantifying T cell activation, CD4 T cells were collected 1 day after anti-CD3/CD28 bead stimulation and then stained with PE-labeled anti-human CD25 antibody (clone M-A251) (BD Biosciences) or PE-labeled anti-human CD69 antibody (clone FN50) (BD Biosciences), respectively. For quantifying CD2, CD3ε, and CXCR4 surface expression on Jurkat T cells, FITC-labeled anti-human CD2 antibody (clone RPA-2.10) (Biolegend), FITC-labeled anti-human CD3ε antibody (clone UCHT1) (BD Biosciences), or PE-labeled anti-human CXCR4 antibody (clone 12G5) (Biolegend), respectively, was used for staining. Surface staining was conducted on ice for 30 minutes in the dark using antibody dosages as recommended by the manufacturers. Cells were washed with cold PBS-0.1% BSA and then analyzed on a FACSCalibur (BD Biosciences).

#### Viral fusion assay

The BlaM-Vpr-based viral fusion assay was performed as previously described (Cavrois et al., 2002). Briefly, viruses were generated by co-transfection of HEK293T cells with three plasmids: pNL4-3 proviral DNA, pCMV4-BlaM-Vpr (kindly provided by Dr. Warner C. Greene), and pAdVAntage (Promega) (in the ratio of 6:2:1). Supernatant was harvested at 48 hours post co-transfection, concentrated, and then used for infection of resting CD4 T cells. For infection of resting CD4 T cells, 400 ng (p24) of HIV-1 (BlaM-Vpr) was used for 2 million cells. As a control, cells were pretreated with 10 μM AMD3100 for 1 hour and then infected with HIV-1 (BlaM-Vpr) virus for 2 hours. Infected cells were washed with medium and resuspended in 100 μl CCF2-AM loading solution, and inculabted for 1 hour at room temperature at dark. Cells were washed with 200 μl of development medium, resuspended into 200 μl of development media and incubated at room temperature for 16 hours in dark, and then washed and resuspended in 200 μl 1.2% paraformaldehyde for flow cytometry using a Becton Dickinson LSR II (Becton Dickinson).

#### Cell fractionation

For fractionating actin-associated HIV-1 DNA, infected resting CD4 T cells were washed and then lysed in ice-cold fractionation lysis buffer (10 mM Tris-Cl, pH 7.5, 140 mM NaCl, 5 mM KCl, 10 mM EDTA, 1% NP-40). For fractionation control, a plasmid DNA, pMSCVneo, was added into the lysates. Cell lysates were centrifuged at 1,000 x *g* for 3 minutes at 4°C to pellet the nucleus. The cytosolic fractions in the supernatants were collected and centrifuged at 14,000 x *g* for 30 minutes. Pellets were resuspended in NTENT buffer (150 mM NaCl, 10 mM Tris-Cl, pH 7.2, 1 mM EDTA, 1% Triton X-100) and centrifuged again at 14,000 x *g* for 30 min. Pellets were resuspended in lysis buffer for DNA extraction (Promega Wizard SV Total DNA Kit, Promega).

#### Real-time PCR quantification of HIV-1 DNA

Quantitative real-time PCR analyses of viral late RT DNA were carried out with the Bio-Rad iQ5 real-time PCR detection system as described previously (Yoder et al., 2008). Briefly, each reaction contained 1 x TaqMan Universal PCR Master Mix (Applied Biosystems), 300 nM each of the primers and 300 nM of the probe. The PCR was carried out at 50°C for 2 minutes, 95°C for 10 minutes, and 40 cycles of 95°C for 15 seconds and 60°C for 60 seconds. The sequences of the primers and probe are: the forward primer 5’LTR-U5 (5’- AGATCCCTCAGACCCTTTTAGTCA-3’), the reverse primer 3’ gag (5’- TTCGCTTTCAAGTCCCTGTTC-3’), and the probe FAM-U5/gag (5'-FAM- TGTGGAAAATCTCTAGCAGTGGCGCC-BHQ-3'). For measuring HIV 2-LTR circular DNA, real-time PCR was conducted with the primers MH535 (5’-AACTAGGGAACCCACTGCTTAAG-3’) and MH536 (5’-TCCACAGATCAAGGATATCTTGTC-3’) and the probe MH603 (5'-FAM- ACACTACTTGAAGCACTCAAGGCAAGCTTT-BHQ-3'), as previously described (Kelly et al., 2008). Briefly, each reaction contained 1 x TaqMan Universal PCR Master Mix (Applied Biosystems), 300 nM each of the primers and 300 nM of the probe. The PCR was carried out at 50°C for 2 minutes, 95°C for 10 minutes, and 40 cycles of 95°C for 15 seconds and 60°C for 60 seconds. The DNA standard used for both late DNA and 2-LTR circle quantification was constructed by using a plasmid containing a complete 2 LTR region (pLTR-2C, cloned by amplification of infected cells with 5’-TGGGTTTTCCAGTCACACCTCAG-3’ and 5’-GATTAACTGCGAATCGTTCTAGC-3’). Measurement was run in triplicate ranging from 1 to 10^6^ copies of pLTR-2C mixed with DNA from uninfected cells.

#### Confocal fluorescent microscopy

For monitoring actin dynamics after stimulation of resting CD4 T cells with anti-CD2 antibody beads, resting CD4 T cells (5 x 10^6^ cells) were electroporated with 5 μg of pLifeAct-EGFP plasmid using Nucleofactor and Nucleofector Kit R (Lonza). Electroporation was carried out as recommended by the manufacturer. Electroporated cells were cultured and treated with anti-CD2 antibody beads (2 beads per cell) at 48 hours post-electroporation. Actin dynamics were monitored by live-cell fluorescence imaging using the UltraView Vox confocal system (PerkinElmer, Co., contains cell culture chamber, Tokai Hit) equipped with a Nikon Eclipse Ti-E microscope with a 60×, 1.4 NA oil-immersion objective lens. The images were captured with an EM-CCD (Hamamatsu C9100-14). Data were analyzed with Volocity 6.3.0. The white field, the green (F-actin) fluorescent field, and the merged field are shown (from left to right).

For confocal imaging of actin polymerization upon SDF-1 stimulation, resting memory CD4 T cells (10^6^ cells) were pretreated with SDF-1 (12.5 nM) for 15 minutes, fixed, permeabilized for 20 minutes at room temperature, washed twice, and then stained with 5 μl of 0.3 mM FITC-labeled phalloidin (Sigma) for 30 minutes on ice in the dark. Cells were stained with DAPI (4′, 6-diamidino-2-phenylindole) for nuclear DNA. Stained cells were imaged using a Zeiss Laser Scanning Microscope, LSM 510 META, with a 40 NA 1.3 or 60 NA 1.4 oil DIC Plan-Neofluar objective. Samples were excited with a laser line, 488 nm for FITC. Images were simultaneously recorded in two channels: channel one, fluorescent emissions from 505 to 530 nm for FITC (green); channel two, DIC.

For confocal imaging of CD2 and CXCR4 cellular localization, Jurkat CD4 T cells (1 x 10^6^ cells) were electroporated with 500 ng of pCMV3-CD2-C-GFPSpark and 500 ng of pCMV3-CXCR4-C-OFPSpark plasmids (SinoBiological) using Nucleofactor and Nucleofector Kit R (Lonza). At 48 hours post-electroporation, cells were stimulated with 200 ng (p24) of single cycle HIV-1(gp160) viral particles for 30 min at 37°C. Following stimulation, cells were kept on ice for confocal imaging. To visualize the cellular localization of CD2-GFP and CXCR4-OFP, cells were imaged using a Carl Zeiss Laser Scanning Microscope Zen 780 (Carl Zeiss, Thornwood, NY) with a Plan-Apochromat 60x/1.4 Oil DIC M27 objective. Laser lines of 488 nm and 564 nm were used to excite GFP and OFP, respectively. Imaging data were exported using Zen software and further processed using Adobe Photoshop.

#### Measurement of phospho-cofilin by western blot and intracellular staining

Resting CD4 T cells were treated with anti-CD2 beads (2 beads per cell) from 0 min to 60 minutes, fixed, and stained with Intracellular Protein Staining Kit (Virongy) for p-cofilin staining. Cells were washed twice and stained with 0.5 μl of Alexa Fluor 488 chicken anti-rabbit antibody (Thermo Fisher Scientific) for 30 min at room temperature. After washing, stained cells were analyzed on FACSCalibur (Becton Dickinson). For western blot, anti-CD2 antibody bead-stimulated resting CD4 T cells were lysed in NuPAGE LDS Sample Buffer (Thermo Fisher Scientific). Cell lysates were analyzed by SDS-PAGE, followed by transferring onto nitrocellulose membranes (Thermo Fisher Scientific) that were blocked for 30 min with 5% milk. Blots were incubated with a rabbit anti-phospho-cofilin (ser3) antibody (1:1000 dilution) (Cell Signaling) overnight at 4˚C, washed, and incubated with a horseradish peroxidase (HRP)-conjugated anti-rabbit IgG antibody (1:2000 dilution) (Cell Signaling). Blots were also stripped and reprobed with a goat anti-GAPDH antibody (1:1000 dilution) (Abcam) (4˚C for overnight), followed by incubation with a horseradish peroxidase-conjugated anti-goat antibody (1:2500 dilution) (KPL). For chemiluminescent detection of HRP-conjugated antibodies, the light signals were captured on a cooled CCD camera (Alpha Innotech) using chemiluminescent SuperSignal West Femto Maximum Sensitivity Substrate (Pierce).

#### Fluorescence resonance energy transfer (FRET) assay

Jurkat CD4 T cells were pre-chilled on ice for 30 min before antibody staining and then incubated with APC-labeled anti-human CD2 antibody (clone RPA-2.10) (BD Biosciences) and PE-labeled anti-human CXCR4 antibody (clone 12G5) (Biolegend) for the detection of FRET between CD2 and CXCR4. For control, cells were incubated with APC-labeled anti-human CD3ε antibody (clone UCHT1) (BD Biosciences) and PE-labeled anti-human CXCR4 antibody (clone 12G5) (Biolegend) for the detection of FRET between CD3ε and CXCR4. Antibody surface staining was conducted on ice for 30 min, followed by washing with cold staining buffer (HBSS with 10 mg/ml BSA and 10 mM HEPES, pH 7.5). The stained cells were then incubated at 37°C with or without 500 ng HIV (NL4-3) viral particles for 30 min to induce FRET signals. Cells were washed with cold staining buffer and then washed with acid-washing buffer (20 mM HCl/HBSS, pH 2.0) on ice for 5-10 min. After washing, the cells were resuspended in cold staining buffer and analyzed immediately for FRET on FACSCalibur (Becton Dickinson).

### Quantification and statistical analysis

Sample analyses and inhibition assays were performed in triplicate, and statistical significance was determined using two-tailed T-Test in Prism 7 (Graph Pad). Significance p values are indicated in figure legends.

## Data Availability

The published article includes all data generated or analyzed during this study. Data reported in this paper will be shared by the lead contact upon request. This paper does not report original code. Any additional information required to reanalyze the data reported in this paper is available from the lead contact upon request.

## Footnotes

Supplemental information can be found online at https://doi.org/10.1016/j.isci.2021.103305.
