# Supplementary material for: Prestimulation of CD2 confers resistance to HIV-1 latent infection in blood resting CD4 T cells
Source: iScience. 2021 Oct 16;24(11):103305. doi: 10.1016/j.isci.2021.103305 (PMC8571718; doi:10.1016/j.isci.2021.103305)
Supplement: Document S1. Figures S1–S8 [file mmc1.pdf]

iScience, Volume *24*

Supplemental information

Prestimulation of CD2 confers resistance to HIV-1 latent infection in blood resting CD4 T cells

Sijia He, Jia Guo, Yajing Fu, Mark Spear, Chaolong Qin, Shuai Fu, Zongqiang Cui, Wenwen Jin, Xuehua Xu, Wanjun Chen, Hong Shang, and Yuntao Wu

SUPPLEMENTAL INFORMATION

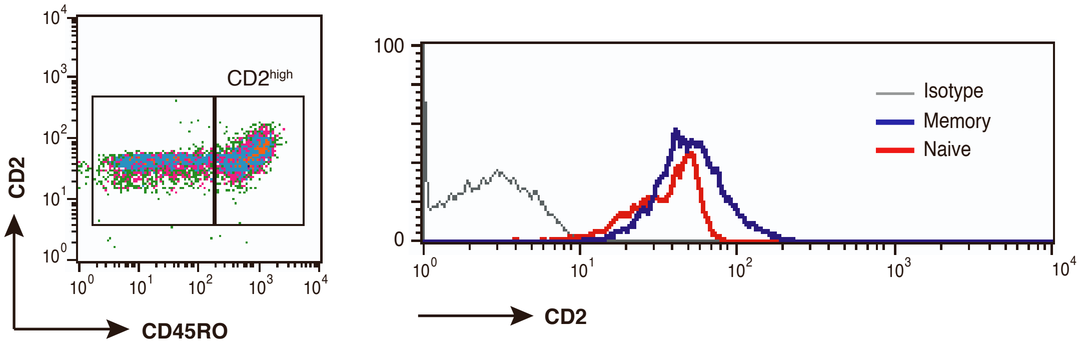

Fig. S1. Expression of CD2 on the surface of human blood resting CD4 T cells, Related to Fig. 1. Blood resting CD4 T cells were stained with FITC-labeled anti-CD2 antibody and co-stained with PE-Cy5-labeled anti-CD45RO antibody, and then analyzed by flow cytometry. Surface expression of CD2 was measured on both resting naïve (CD45RO$^-$) and memory (CD45RO$^+$) CD4 T cells. Memory CD4 T cells express higher levels of CD2.

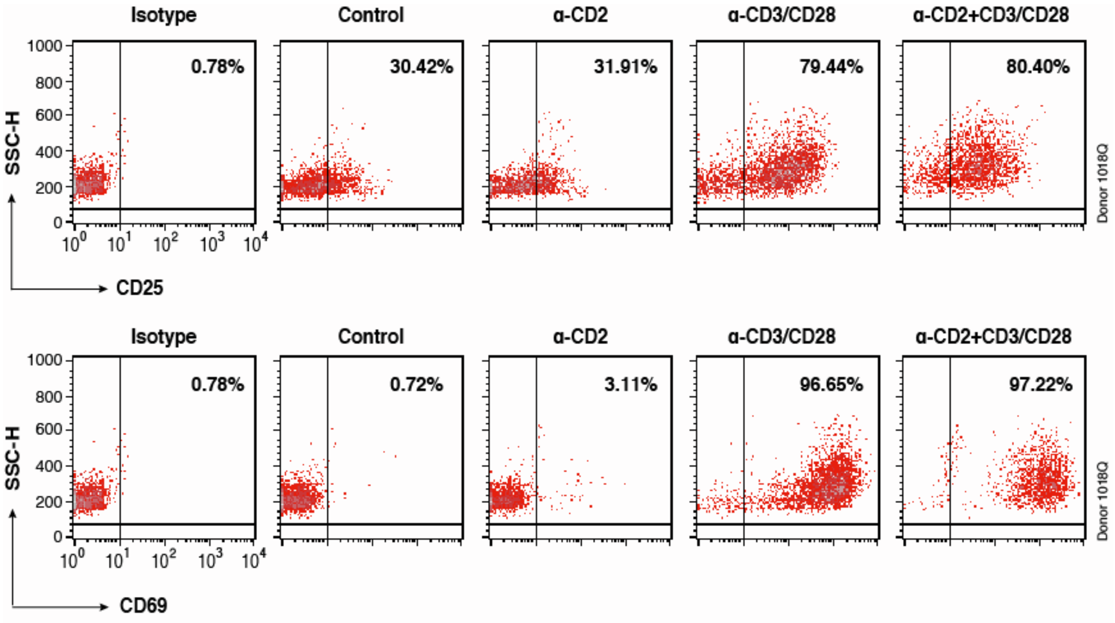

Fig. S2. CD2 stimulation does not inhibit T cell activation, Related to Fig. 1. Blood resting CD4 T cells were stimulated with or without α-CD2 beads (2 beads per cell) overnight and then cultured for an additional 5 days. Cells were activated with α-CD3/CD28 beads (4 beads per cell). One day after T cell activation, cells were stained with PE-labeled anti-CD25 antibody or PE-labeled anti-CD69 antibody and analyzed by flow cytometry.

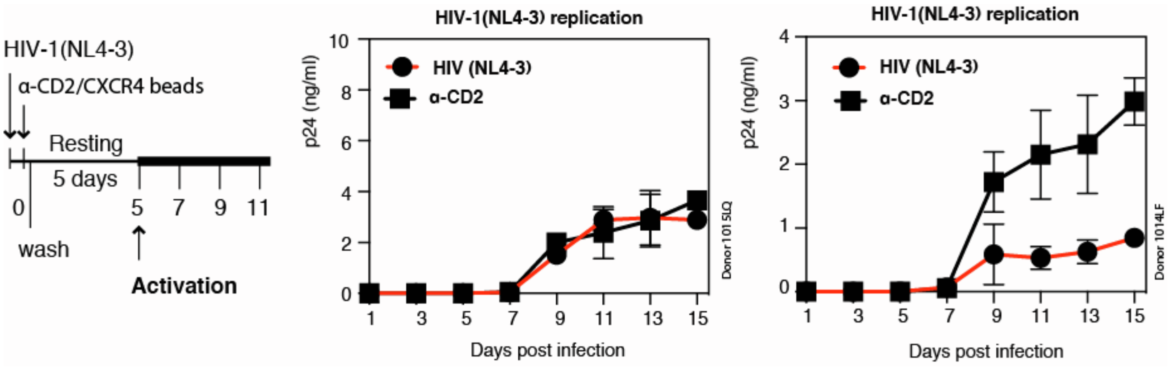

Fig. S3. CD2 poststimulation did not inhibit HIV-1 latent infection of resting CD4 T cells, Related to Fig. 1. Blood resting CD4 T cells were infected with HIV-1(NL4-3) for 2 hours, washed, and then stimulated with α-CD2 beads (2 beads per cell). Cells were cultured for 5 days and then activated with α-CD3/CD28 beads (4 beads per cell). Viral replication was monitored by p24 release.

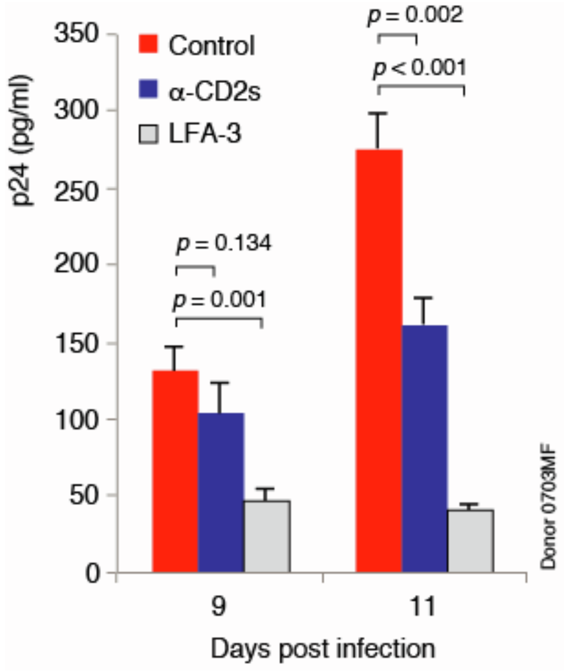

Fig. S4. Inhibition of HIV-1 replication by soluble LFA-1 and anti-CD2 antibody, Related to Fig 1. Resting CD4 T cells were prestimulated with soluble α-CD2 antibody (α-CD2s, 2 μg/ml) or recombinant human LFA-3 (2 μg/ml) for 1 hour, and then infected with HIV-1(NL4-3). Cells were washed, cultured, and then activated at day 5 with anti-CD3/CD28 beads (4 beads per cell). Viral replication was measured by p24 release. Inhibition assays were performed in triplicate, and data are represented as mean $\pm$ SEM. Statistical significance was determined using two-tailed T-Test in Prism 7 (Graph Pad). Significance p values are indicated.

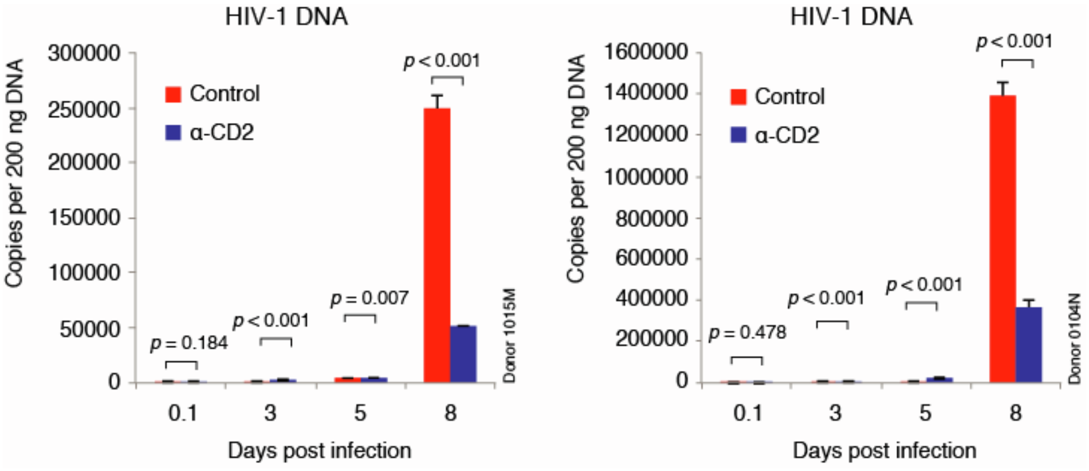

Fig. S5. CD2 prestimulation inhibits HIV-1 DNA accumulation in resting CD4 T cells, Related to Fig. 2. Resting CD4 T cells were prestimulated or not with α-CD2 beads (2 beads per cell), infected with HIV-1(NL4-3) **(**using an equal p24 level), washed, cultured for 5 days, and then activated with α-CD3/CD28 beads. Intracellular HIV late DNA was quantified by real-time PCR at indicated time points using an equal amount of total cellular DNA (200 ng). Samples were analyzed in triplicate, and data are represented as mean + SEM. Statistical significance was determined using two-tailed T-Test in Prism 7 (Graph Pad). Significance p values are indicated.

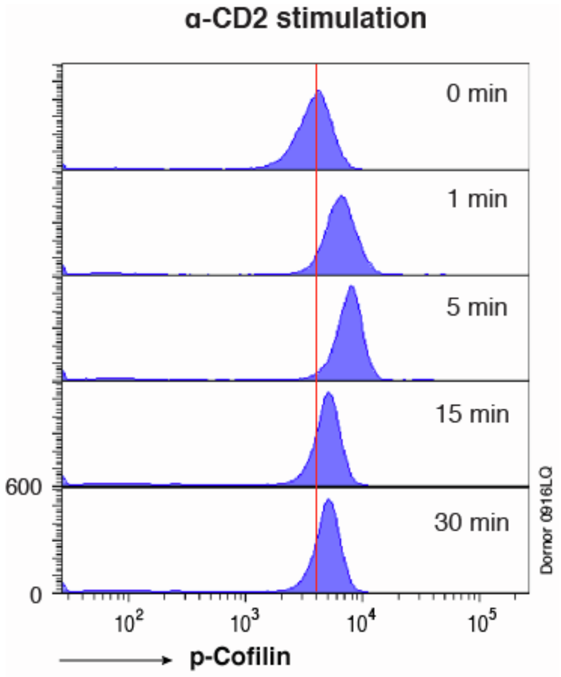

Fig. S6. CD2 stimulation triggers cofilin activation, Related to Fig. 3. Blood resting CD4 T cells were treated with α-CD2 beads (2 beads per cell) for different time courses from 0 min to 60 minutes, fixed, and then permeabilized for intracellular staining with an anti-p-cofilin antibody. The histogram shows p-cofilin staining detected with flow cytometry.

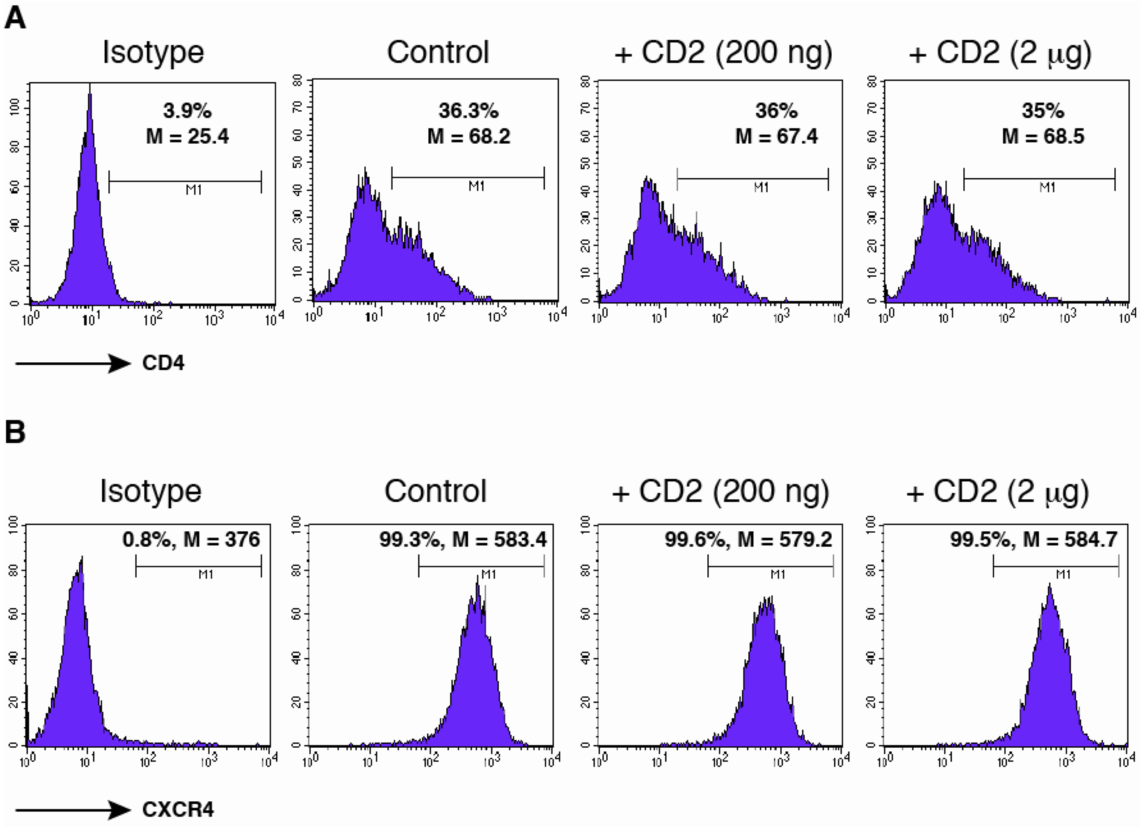

Fig. S7. CD2 stimulation did not lead to downregulation of CD4 or CXCR4, Related to Fig. 4. Jurkat CD4 T cells were treated with the indicated doses of soluble CD2 antibody or with a control mouse IgG1k antibody for 1 hour. Cells were stained with PE-labeled anti-CD4 antibody (**A**) or PE-labeled anti-CXCR4 antibody (**B**) and analyzed by flow cytometry.

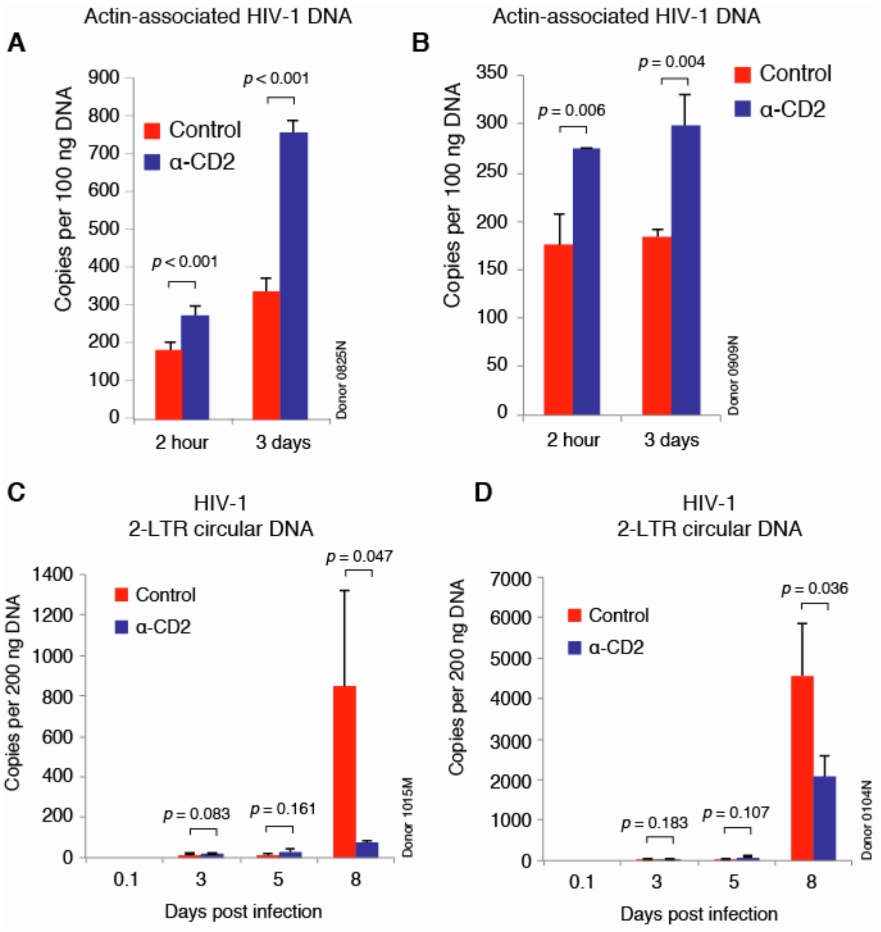

Fig. S8. Effects of CD2 prestimulation on actin-associated HIV-1 DNA synthesis and viral 2-LTR circular DNA synthesis, Related to Fig 5. (A and **B**) Actin-associated HIV DNA synthesis after prestimulation with α-CD2 beads. Resting CD4 T cells were prestimulated with α-CD2 beads and infected with HIV-1(gp160) (using an equal p24 level). Cytoskeleton was fractionated by ultracentrifuge, and actin-associated viral DNA was quantified by real-time PCR using an equal amount of total cellular DNA (100 ng). (**C** and **D**) HIV-1 2-LTR circle synthesis after α-CD2 prestimulation. Resting CD4 T cells were prestimulated with α-CD2 beads and infected with HIV-1(gp160) (using an equal p24 level). Cells were activated at day 5 with α-CD3/CD28 beads. Total cellular DNA was purified, and HIV-1 2-LTR circular DNA was quantified by real-time Q-PCR using an equal amount of total cellular DNA (200 ng). Samples were analyzed in triplicate, and data are represented as mean ± SEM. Statistical significance was determined using two-tailed T-Test in Prism 7 (Graph Pad). Significance p values are indicated.

Movie S1

Stimulation with anti-CD2-beads triggers actin polymerization around the bead-CD4 T cell junction, Related to Fig. 3. Blood resting CD4 T cells were purified, electroporated with pLifeAct-EGFP, and then stimulated with α-CD2 beads. Actin polymerization was monitored by live-cell fluorescence imaging microscopy, which uses the UltraView Vox confocal system (PerkinElmer, Co., contains cell culture chamber, Tokai Hit) with a Nikon Eclipse Ti-E microscope with a 60×, 1.4 NA oil-immersion objective lens. The images were captured with an EM-CCD (Hamamatsu C9100-14). Data were analyzed with Volocity 6.3.0. The green (F-actin) fluorescent field, the white field, and the merged field are shown (from left to right).
